# Applying vibration in early postmenopausal osteoporosis promotes osteogenic differentiation of bone marrow-derived mesenchymal stem cells and suppresses postmenopausal osteoporosis progression

**DOI:** 10.1042/BSR20191011

**Published:** 2019-09-03

**Authors:** Huiming Li, Wenchao Wu, Xueling He, Chengjian Cao, Xiaoqin Yu, Ye Zeng, Liang Li

**Affiliations:** 1 Institute of Biomedical Engineering, West China School of Basic Medical Science and Forensic Medicine, Sichuan University, Chengdu 610041, Sichuan, P.R. China; 2 Laboratory of Cardiovascular Diseases, Regenerative Medicine Research Center, West China Hospital, Sichuan University, Chengdu 610041, P.R.China; 3 Laboratory Animal Center, Sichuan University, Chengdu 610041, Sichuan, P.R. China

**Keywords:** ERα, low magnitude vibration, postmenopausal osteoporosis, rat bone marrow-derived mesenchymal stem cells, Wnt

## Abstract

We aimed to evaluate whether applying low magnitude vibration (LMV) in early postmenopausal osteoporosis (PMO) suppresses its progression, and to investigate underlying mechanisms. Rats were randomly divided into Sham (Sham-operated), Sham+V, OVX (ovariectomized), OVX+E2 (estradiol benzoate), OVX+V (LMV at 12–20 weeks postoperatively), and OVX+Vi (LMV at 1–20 weeks postoperatively) groups. LMV was applied for 20 min once daily for 5 days weekly. V rats were loaded with LMV at 12–20 weeks postoperatively. Vi rats were loaded with LMV at 1–20 weeks postoperatively. Estradiol (E2) rats were intramuscularly injected at 12–20 weeks postoperatively once daily for 3 days. The bone mineral densities (BMDs), biomechanical properties, and histomorphological parameters of tibiae were analyzed. *In vitro*, rat bone marrow-derived mesenchymal stem cells (rBMSCs) were subjected to LMV for 30 min daily for 5 days, or 17β-E2 with or without 1-day pretreatment of estrogen receptor (ER) inhibitor ICI 182,780 (ICI). The mRNA and protein expresion were performed. Data showed that LMV increased BMD, bone strength, and bone mass of rats, and the effects of Vi were stronger than those of E2. *In vitro*, LMV up-regulated the mRNA and protein expressions of Runx2, Osx, Col I, and OCN and down-regulated PPARγ, compared with E2. The effects of both LMV and E2 on rBMSCs were inhibited by ICI. Altogether, LMV in early PMO suppresses its progression, which is associated with osteogenic differentiation of rBMSCs via up-regulation of ERα and activation of the canonical Wnt pathway. LMV may therefore be superior to E2 for the suppression of PMO progression.

## Introduction

Postmenopausal osteoporosis (PMO) usually occurs 5–10 years after menopause, and is mainly caused by a decrease in estrogen. It is a type of systemic skeletal disease characterized by osteopenia, deterioration of bone microarchitecture, increased bone fragility, and susceptibility to fracture [1]. With the aging global population, the incidence of PMO is rapidly rising. PMO seriously influences patients’ health and quality of life, placing a heavy burden on patients’ families and the national economy [2]. Osteoporotic fractures have become a public health problem. It is estimated that 84% of patients with osteoporotic fractures did not receive a clear clinical diagnosis before the fracture occurred, creating a missed opportunity for treatment to regulate bone metabolism and to improve osteoporosis at an early stage of PMO [3]. Therefore, early detection of bone mass damage and intervention is key to prevent or delay the occurrence of osteoporotic fracture [4,5].

Currently, the strategies of prevention and treatment of PMO primarily include diet therapy, drug therapy, and exercise therapy. Exercise therapy can promote bone formation and inhibit bone resorption, exceeding most drugs [6]. As a measure that is noninvasive, has few side effects, and is highly tolerated, exercise therapy has become more and more accepted by doctors and patients. Low magnitude vibration (LMV) is a type of exercise therapy, and is defined as vibration with an acceleration of gravity of less than 1×***g*** (***g*** = 9.81 m/s^2^) [7]. LMV has been found to stimulate muscle and bone tissue metabolism [8], increase bone mineral density (BMD), promote bone formation, and inhibit bone resorption and bone loss [9–12]. In addition, it can increase muscle strength by increasing neuromuscular activity, improving balance function and coordination, and reducing the risk of fall and fracture [13], and may also increase blood circulation in muscle and bone tissue, thereby increasing the nutrient supply to bone tissue [14]. Because of its low load and simple operation, LMV is especially suitable for certain groups of osteoporosis patients, such as elderly patients, drug-intolerant patients, and long-term bedridden patients [6]. Accordingly, LMV has important clinical application value and broad development prospects in the treatment of PMO and prevention of age-related bone loss [14–16]. However, it remains unclear whether applying LMV in the early stages of PMO could suppress progression.

In our previous research, we found that in ovariectomized osteoporosis rats, not only was osteoblast and osteoclast function changed, but also the osteogenic differentiation of rat bone marrow-derived mesenchymal stem cells (rBMSCs). This is likely an important factor affecting the occurrence and development of PMO. BMSCs are common precursor cells of osteoblasts and adipocytes. In a study on ovariectomized osteoporosis rats, the osteogenic differentiation of BMSCs was decreased, but the adipogenic differentiation of BMSCs was increased [17]. The specific mechanism remains unclear.

In another study on PMO, we found decreased responsiveness of bone tissue cells to mechanical stimulation after estrogen level reduction. Biaxial tensile strain *in vitro* can up-regulate the expression of estrogen receptor (ER) α (ERα) and β-catenin in rBMSCs, and ERα can functionally interact with β-catenin [18]. ER, especially ERα, is the most important element of the estrogen-mediated effect, and is expressed in both osteoblasts and osteoclasts, as well as in their precursors. ERα not only mediates the estrogen effect in bone cells, but also participates in the transmission of mechanical signals, and plays an important role in bone formation and metabolism. Osteoblast-like cells from ERα knockout mice have deficient responses to mechanical strain, and have a less osteogenic response to loading [19]. In addition, proliferation of BMSCs was inhibited by ER blockade [20], suggesting that ERα is an important mechanical receptor. β-catenin is one of the most important factors in the canonical Wnt pathway, which regulates the osteogenic differentiation of BMSCs and participates in the response of BMSCs to mechanical stimuli. Whether LMV regulates the osteogenic differentiation and adipogenic differentiation of BMSCs via ERα-canonical Wnt pathway remains unclear.

In the present study, we evaluated the effects of LMV (0.9×***g*** and 45 Hz) on ovariectomized rats at 1 or 12 weeks postoperatively, and investigated the effects of LMV on osteogenic differentiation of rBMSCs and ERα signaling in rats. The effects of vibration and estradiol (E2) were also compared.

## Materials and methods

### Animal model and experimental design

A total of 60 3-month-old female virgin Sprague–Dawley rats (average body weight, 265 ± 15 g) were obtained from the Laboratory Animal Center of Sichuan University, Chengdu, China. The rats for the experiments were housed in the animal care facility in a standard breeding environment with a room temperature of 25°C and humidity of 55% with *ad libitum* access to food and water. Acclimatized for 1 week, rats were randomly divided into six groups (*n*=10 for each group): (1) Sham group, in which adipose tissue equivalent to two ovaries was removed from rats, followed by feeding for 20 weeks; (2) Sham+V group, in which rats were subjected to whole-body vertical vibration stimulation from 12 to 20 weeks after sham operation; (3) OVX group, in which rats underwent bilateral ovariectomy, followed by feeding for 20 weeks; (4) OVX+E2 group, in which rats were intramuscularly injected by E2 benzoate (0.5 mg/kg) once every 3 days from 12 to 20 weeks after ovariectomy; (5) OVX+V group, in which rats were subjected to vibration stimulation from 12 to 20 weeks after ovariectomy; and (6) OVX+Vi group, in which rats were subjected to vibration stimulation from 1 to 20 weeks after ovariectomy. The vibration was performed by using the vibration device with a vibration box (ZD-50T, Guangzhou Meiyifeng Test Equipment Co., Ltd, China) at 0.9×***g*** and 45 Hz for 20 min everyday, once daily, for 5 days a week.

Rats were weighed every 4 weeks during the experiment, and all animals received hypodermic injections of 10 mg/kg Calcein (Sigma, U.S.A.) on days 126, 127, 136, and 137 postoperatively to label bone forming surfaces. At the end of the experiment, rats were killed by cervical dislocation. Each uterus was extracted and weighed. The bilateral tibiae were cleaned off skin, muscle, and tendons. Left tibiae were wrapped in saline-soaked gauze and stored at −20°C for BMD detection and three-point bending tests, while right tibiae were fixed overnight in 4% paraformaldehyde and then placed in 70% ethanol for histomorphometric analysis. All the experimental procedures were approved by the Ethics Committee of Sichuan University. All experiments were performed at Institute of Biomedical Engineering, West China School of Basic Medical Science and Forensic Medicine of Sichuan University.

### BMD detection

The left tibiae were scanned with the VivaCT80 micro-computed tomography system (Scanco Medical, Switzerland). The scanning parameters were 70 kV and 113 μA. The samples were scanned with high resolution, and each layer was scanned at a thickness of 19 μm. After completing the overall scan of the tibia, we selected a ‘region of interest’ (ROI) at 1.5 mm below the upper growth plate, then 3 mm below the first point for 3D reconstruction. Then, the 3D structure images were automatically evaluated for BMD using the μCT Tomography software (V6.3, Scanco Medical) .

### Bone biomechanical test

Biomechanical properties of left tibiae were evaluated by three-point bending test using a universal material test machine (AGIS-201, SHIMADZU, Japan). The two ends of the tibia were placed on a special bracket with a span of approximately 35 mm so that they did not move during the experiment. A cylindrical indenter moved down to the point of 5 mm from the proximal end of the tibia with a continuous displacement of 1 mm/min until the metaphyseal tibia broke. Then, the damaged part of the metaphyseal tibia was removed, and the rest of the diaphyseal tibia was placed on two cylindrical brackets with a span of 20 mm. A cylindrical indenter moved down to the midpoint of the diaphyseal tibia with a continuous displacement of 1 mm/min until the diaphyseal tibia broke. From the Force-Stroke curve, biomechanical parameters including elastic modulus, maximum stress, and failure stress were determined.

### Bone histomorphometric analysis

Right tibiae were dehydrated by sequential ascending concentrations of ethanol and degreased by xylene. The metaphysis and diaphysis of the tibiae were embedded in methyl methacrylate (Sigma, U.S.A.), respectively. The embedded metaphyseal tibia specimens were cut longitudinally into sections of 5 μm along the sagittal plane using a hard tissue microtome (RM 2155, Leica, Germany). The sections were stained with Masson–Goldner Trichrome for measuring static parameters. Starting at the junction of the tibia and fibula, and toward the proximal end of the tibia, the diaphyseal tibiae were sawed transversely into 100-μm slices, and the second slices were taken and ground into 40–5 μm slices, observed with a fluorescence microscope (MGC30, Leica, Germany).

A semi-automatic image analysis system (OsteoMeasure, OsteoMetrics, U.S.A.) was used to quantitatively measure the secondary cancellous bone between 1 and 4 mm below the epiphyseal line of the metaphyseal tibia and the cortical bone of the diaphyseal tibia.

### rBMSCs isolation, culture, and grouping

rBMSCs were obtained from 4-week-old female Sprague–Dawley rats (average body weight of 90 ± 10 g, the Laboratory Animal Center of Sichuan University, Chengdu, China). Briefly, the bone marrow of the tibias and femurs was flushed out with DMEM-LG basal medium (Gibco, U.S.A.), centrifuged, and resuspended with DMEM-LG complete medium containing 85% DMEM-LG medium, 15% fetal bovine serum (FBS) (Sigma, U.S.A.), and 1% penicillin–streptomycin solution (HyClone, U.S.A.), and was cultured in a humidified incubator with 5% CO_2_ at 37°C. The medium was replaced every 3 days, and adherent cells were passaged at 70–80% confluence. The third passage cells were divided into five groups for experiments: (1) control group, in which cells were cultured in DMEM-LG complete medium for 6 days; (2) E2 group, in which cells were cultured in DMEM-LG complete medium for 1 day, then cultured in DMEM-LG complete medium containing 100 nM 17β-E2 (Sigma, U.S.A.) for 5 days; (3) V group, in which cells were cultured in DMEM-LG complete medium for 1 day, and then subjected to vibration of 0.9×***g***, 45 Hz, and 30 min/day for 5 days; (4) ICI+E2 group, in which cells were cultured in DMEM-LG complete medium containing 50 ng/ml ICI 182,780 (ER inhibitor) (MCE, U.S.A.) for 1 day, then cultured in DMEM-LG complete medium containing 100 nM 17β-E2 for 5 days; and (5) ICI+V group, in which cells were cultured in DMEM-LG complete medium containing 50 ng/ml ICI 182,780 for 1 day, and then cultured in DMEM-LG complete medium, simultaneously subjected to vibration of 0.9×***g***, 45 Hz, and 30 min/day for 5 days.

### Real-time quantitative RT-PCR

Total RNA was isolated from cells of each group using TRIzol reagent (Invitrogen, U.S.A.), and reverse transcribed to cDNA using PrimeScript™ RT reagent Kit (TaKaRa, China). A quantitative RT-PCR assay was performed on a CFX96™ Real-Time System (Bio-Rad, U.S.A.) using specific primers and SYBR® Premix ExTaq™ Ⅱ (TaKaRa, China). Primers were designed as shown in Table 1. Relative expression values were calculated using the comparative threshold cycle (2^−ΔΔ*C*^_T_) [21]. *GAPDH* served as the housekeeping gene.

**Table 1:** Primer sequences of qRT-PCR

Genes	Forward (5′–3′)	Reverse (5′–3′)	Product size (bp)
*GAPDH*	CTTTGGTATCGTGGAAGGACTC	GTAGAGGCAGGGATGATGTTCT	132
*Runx2*	GATGAGAACTACTCTGCCGAGCTAC	CAAAGTGAAACTCTTGCCTCGTC	117
*Osx*	AGCTGCCTACTTACCCGTCTGA	TGCCCACTATTGCCAACTGC	136
*Col I*	CCTGAGCCAGCAGATTGAGAA	GCAGCCTTGGTTAGGGTCGA	136
*OCN*	ACCCTCTCTCTGCTCACTCTGCT	GCTCCAACTCCATTGTTGAGGTAG	164
*ER*α	CCTGTTTGCTCCTAACTTGCTCT	TCATCATGCGGAATCGACTTG	110
*Wnt3a*	GAACCGTCACAACAATGAGGCT	AGCAGGTCTTCACTTCGCAACT	104
β*-catenin*	GACTCTGAGAAACTTGTCCGATGC	CCACTTGGCACACCATCATCT	176
*PPAR*γ	CGGTTGATTTCTCCAGCATTTC	GCAGGCTCTACTTTGATCGCACT	133
*RANKL*	CATCGGGTTCCCATAAAGTCAGT	GCAAATGTTGGCGTACAGGTAATA	137
*OPG*	ATTGGCTGAGTGTTCTGGTGGA	CTGGAAAGTTTGCTCTTGCGA	104

### Western blotting

Cells were washed with iced PBS and lysed in RIPA lysis buffer (Beyotime, China). Whole cell proteins were quantified by the BCA Protein Assay Kit (Beyotime, China), separated by 10% SDS/PAGE, and then transferred on to PVDF membrane (Millipore, U.S.A.) and blocked with Tris-buffered saline buffer with Tween 20 (TBST) containing 5% non-fat milk powder. Membranes were incubated with primary antibodies overnight at 4°C, washed with TBST, and incubated with secondary antibodies conjugated with horseradish peroxidase (Boster, China) for 2 h at room temperature. Primary antibodies used in the present study included those against β-actin, ERα, Wnt3a, and β-catenin (Proteintech, U.S.A.). Immunoreactive bands were visualized with an enhanced luminol-based chemiluminescent substrate (ECL) Kit (Beyotime, China) and quantified using ImageJ Plus software (National Institutes of Health, U.S.A.), normalized by β-actin.

### Statistical analysis

All data were analyzed by SPSS 20.0 software (IBM Corp, Armonk, NY). Each test was repeated at least three times, and data were expressed as mean ± standard deviation (SD). Student’s *t* test was used for comparison between two groups or one-way analysis of variance (ANOVA) for multiple groups, followed by Student–Newman–Keuls (S-N-K) test. *P*<0.05 was considered as statistically significant.

## Results

### LMV increased the BMD in both Sham and OVX rats

**Figure 1:**
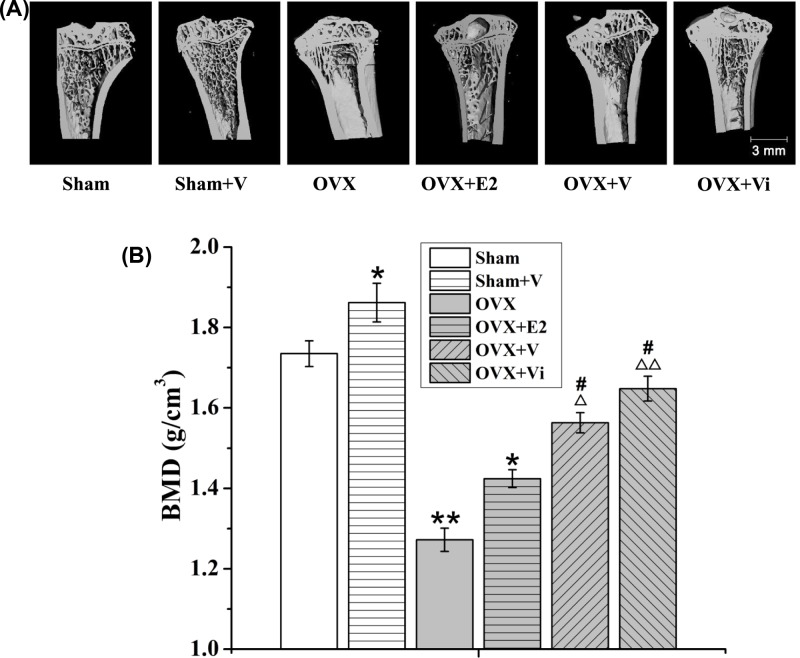
Effect of LMV or E2 on BMD in rats BMD of the left tibiae in each group were measured by microCT. (**A**) Sectional views of the left tibiae in each group. (**B**) BMD of the left tibiae in each group. *P<0.05, **P<0.01 vs. Sham group. ^Δ^*P*<0.05, ^ΔΔ^*P*<0.01 vs. OVX group. ^#^*P*<0.05 vs. OVX+E2 group; (*n*=10).

The BMD of the left tibiae in ovariectomized osteoporosis (OVX) rats was significantly reduced by 26.7% as compared with the sham-operated (Sham) rats (*P*<0.01, Figure 1A,B). LMV (vibration from 12 to 20 weeks after the operation, V) significantly increased the BMD in both Sham and OVX rats, and the increase by V in OVX rats was significantly higher than by E2 (treated from 12 to 20 weeks after the operation). Interestingly, when loading the vibration after operation immediately (vibration from 1 to 20 weeks after ovariectomy, Vi), the BMD was significantly more than the V and E2 groups at the end time points (20 weeks, Figure 1), suggesting strong protection from bone loss by vibration, which was more effective during the early stage of osteoporosis.

### Uterine weight unchanged after LMV and increased after E2

**Figure 2:**
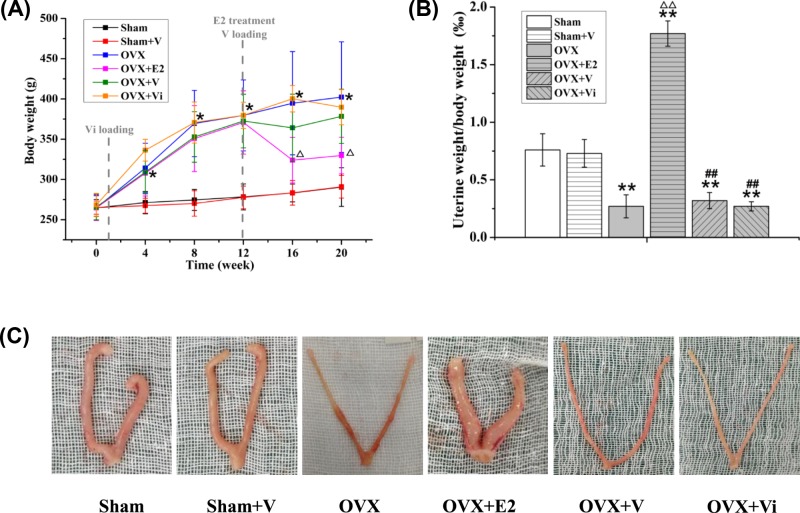
Effect of LMV or E2 on body and uterus weight in rats (**A**) Change in body weights of rats in each group during the experiment. (**B**) The ratio of uterine weight to body weight of rats in each group. *P<0.05, **P<0.01 vs. Sham group. ^Δ^*P*<0.05,^ ΔΔ^*P*<0.01 vs. OVX group. ^##^*P*<0.01 vs. OVX+E2 group (*n*=10). (**C**) Uteri structure of rats in each group.

The body weight of the OVX rats was significantly increased compared with the Sham rats (Figure 2A). After E2 treatment for 4 weeks (from 12 to 16 weeks postoperatively, the average body weight of OVX rats was significantly decreased 17.9% (*P*<0.05). Significant changes in body weight were not observed in rats loaded with V and Vi (Figure 2A).

Ovariectomy significantly resulted in 64.5% decrease in the uterus-to-body weight ratio (*P*<0.01, Figure 2B), with obvious atrophy of the uterus (Figure 2C). The uterus-to-body weight ratio in OVX rats was significantly increased 555.6% (*P*<0.01), and uteri were enlarged by E2 treatment, but did not change by V and Vi (Figure 2B,C). These results suggested that E2 reduced the body weight and enlarged the uteri in OVX rats, but V and Vi did not.

### LMV improved biomechanical properties of the tibiae

**Figure 3:**
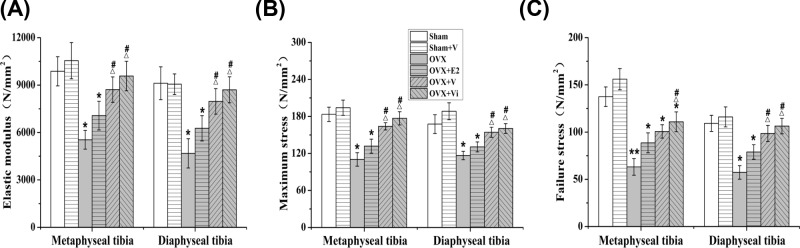
Effect of LMV or E2 on biomechanical parameters in rats The biomechanical parameters, including (**A**) elastic modulus, (**B**) maximum stress, and (**C**) failure stress of metaphyseal tibiae and diaphyseal tibiae, were evaluated by a three-point bending test. *P<0.05, **P<0.01 vs. Sham group. ^Δ^*P*<0.05 vs. OVX group. ^#^*P*<0.05 vs. OVX+E2 group; (*n*=10).

The biomechanical properties of tibiae were analyzed by three-point bending test (Figure 3). The elastic modulus, maximum stress, and failure stress of the metaphyseal and diaphyseal tibiae were significantly reduced in OVX rats compared with Sham rats. The elastic modulus, maximum stress, and failure stress in OVX rats were significantly increased by V and Vi. Compared with E2, V and Vi loading resulted in a significant increase in the elastic modulus and maximum stress of tibiae and the failure stress of diaphyseal tibiae (Figure 3), suggesting that LMV improved the biomechanical properties of bones in OVX rats significantly compared with E2.

### LMV protected the morphological properties of the metaphyseal tibia

**Figure 4:**
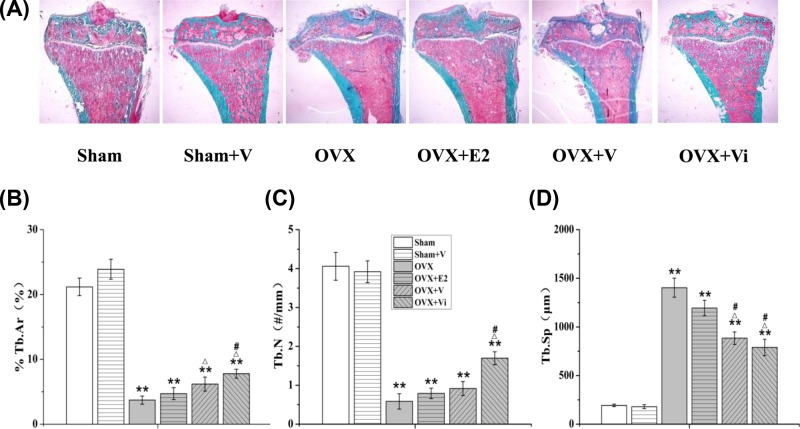
Effect of LMV or E2 on the morphology of cancellous bone in metaphyseal tibiae (**A**) Undecalcified bone sections, Masson–Golder Trichrome: red is the bone marrow, and blue is the bone substance (10×). (**B**–**D**) Changes of histomorphological static parameters including %Tb.Ar, Tb.N, and Tb.Sp in metaphyseal tibiae. **P<0.01 vs. Sham group. ^Δ^*P*<0.05 vs. OVX group. ^#^*P*<0.05 vs. OVX+E2 group; (*n*=10).

Figure 4A–D shows morphological changes in cancellous bone in the metaphyseal tibiae. The percentages of the trabecular area (%Tb.Ar, Figure 4B) and trabecular number (Tb.N, Figure 4C) of the OVX rats were significantly decreased 82.5% (*P*<0.01) and 85.6% (*P*<0.01), respectively, compared with the Sham rats, while the trabecular separation (Tb.Sp) was significantly increased 624.5% (*P*<0.01, Figure 4D). V loading significantly increased the %Tb.Ar 67.0% (*P*<0.05) and reduced the Tb.Sp 37.0% (*P*<0.05). Vi loading significantly reversed the changes in all three morphological parameters in the OVX rats. A significant role of E2 treatment was not observed. These results suggested that the application of LMV in early PMO could effectively reduce the loss of cancellous bone mass in the metaphyseal tibia.

### LMV increased bone mass and promoted bone formation in diaphyseal tibiae

**Figure 5:**
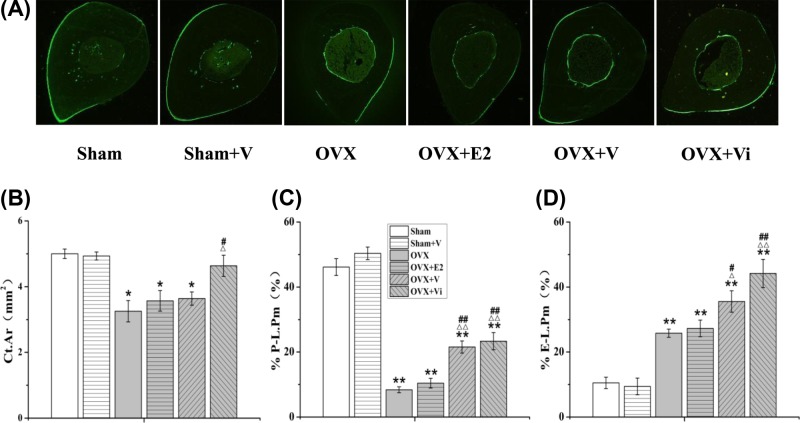
Effect of LMV or E2 on morphology and bone formation in diaphyseal tibiae (**A**) Undecalcified bone ground sections, unstained: green fluorescence indicates bone formation (10×). (**B**–**D**) Changes of histomorphological parameters, including Ct.Ar, %P-L.Pm, and %E-L.Pm in diaphyseal tibiae. *P<0.05, **P<0.01 vs. Sham group. ^Δ^*P*<0.05, ^ΔΔ^*P*<0.01 vs. OVX group. ^#^*P*<0.05, ^##^*P*<0.01 vs. OVX+E2 group; (*n*=10).

Figure 5A–D shows changes in morphology and bone formation in diaphyseal tibiae. Ovariectomy enlarged the bone marrow cavities of rats (Figure 5A), decreased the cortical areas (Ct.Ar, 34.9%, *P*<0.05) and percentage of the labeled periosteal perimeter (%P-L.Pm, 81.8%, *P*<0.01) (Figure 5B,C), and increased the percentage of labeled endosteal perimeter (%E-L.Pm, 145.7%, *P*<0.01) (Figure 5D). V loading significantly increased %P-L.Pm (156.8%, *P*<0.01) and %E-L.Pm (37.8%, *P*<0.05) in OVX rats. Vi loading significantly increased (42.5%, *P*<0.05), %P-L.Pm (178.1%, *P*<0.01) and %E-L.Pm (71.2%, *P*<0.01) in the OVX rats (Figure 5B–D). E2 had no significant effect on morphology and bone formation in the diaphyseal tibiae. These results suggested that LMV promoted the formation of the periosteum and endosteum, and the application of LMV in early PMO could reduce the loss of cortical bone mass in the diaphyseal tibiae.

### Vibration promoted osteogenic differentiation but inhibited adipogenic differentiation of rBMSCs via ERα

**Figure 6:**
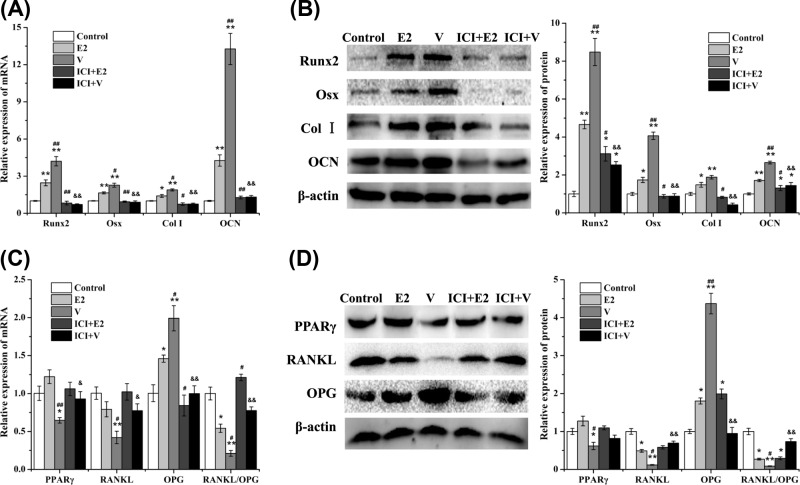
Effect of LMV or E2 on mRNA expression in rBMSCs The changes of mRNA expression in rBMSCs were detected by qRT-PCR, including (**A**) osteogenesis-related genes *Runx2, Osx, Col I*, and *OCN*, and (**C**) adipogenesis-related gene *PPARγ* and osteoclastogenesis-related genes *RANKL, OPG*, and *RANKL/OPG*. The protein levels of (**B**) Runx2, Osx, Col I, and OCN, and (**D**) PPARγ, RANKL, OPG, and RANKL/OPG were performed by Western blotting. *P<0.05, **P<0.01 vs. Control group. ^#^*P*<0.05, ^##^*P*<0.01 vs. E2 group. ^&^*P*<0.05, ^&&^*P*<0.01 vs. V group; (*n*=3).

rBMSCs were isolated and treated with vibration (V) and E2, respectively (Figure 6). The qRT-PCR and Western blotting results showed that osteogenesis-related molecules including Runx2, Osx, Col I, and OCN in the E2 and V groups were significantly increased compared with control, and the effect in V group was more obvious (Figure 6A,B). The mRNA and protein expressions of adipogenesis-related molecule PPARγ in the V group were significantly decreased (*P*<0.05, Figure 6C,D). Moreover, osteoclastogenesis-related molecule RANKL was significantly decreased in the V group (*P*<0.05, Figure 6C,D) but did not significantly change by E2. Both E2 and V significantly increased the mRNA and protein expressions of osteoprotegerin (OPG) (Figure 6C,D). The ratio of RANKL/OPG mRNA and protein in the E2 and V groups were significantly decreased, and the effect in V group was more obvious (Figure 6C,D). These results suggested that vibration promoted the osteogenic differentiation of rBMSCs, inhibited the adipogenic differentiation of rBMSCs, and regulated the osteoclastogenesis, while E2 promoted the osteogenic differentiation but did not inhibit the adipogenic differentiation of rBMSCs, indicating the vibration is a better treatment than E2.

To investigate the role of ERs, ERα was blocked by ER inhibitor ICI (Figure 6). ICI significantly abolished the effect of vibration and E2 on the expression of osteogenesis-related genes, adipogenesis-related genes, and osteoclastogenesis-related genes, suggesting that vibration promoted the osteogenic differentiation of rBMSCs, inhibited the adipogenic differentiation of rBMSCs, and regulated the osteoclastogenesis via ERα.

### Vibration up-regulated the expressions of ERα, Wnt3a, and β-catenin

**Figure 7:**
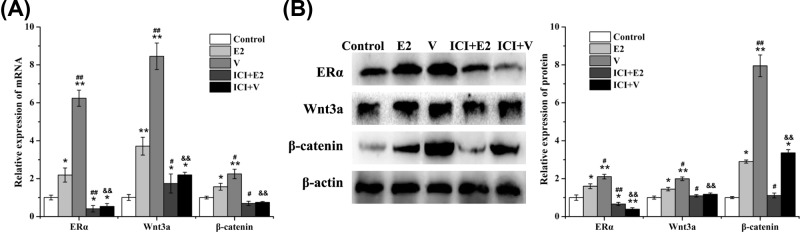
Effect of LMV or E2 on ERα, Wnt3a, and β-catenin expression in rBMSCs (**A**) The changes of mRNA expression in rBMSCs were detected by qRT-PCR, including *ER*α, *Wnt3a*, and *β-catenin*. (**B**) The changes of protein expression in rBMSCs were detected by Western blotting, including ERα, Wnt3a, and β-catenin. *P<0.05, **P<0.01 vs. Control group. ^#^*P*<0.05, ^##^*P*<0.01 vs. E2 group. ^&&^*P*<0.01 vs. V group; (*n*=3).

The expression of ERα, Wnt3a, and β-catenin was detected by qRT-PCR (Figure 7A) and Western blotting (Figure 7B). E2 and V significantly increased the mRNA and protein expression of ERα, Wnt3a, and β-catenin in rBMSCs, and V showed a more obvious effect. Moreover, the ER inhibitor ICI suppressed the effects of E2 and V on the expression of ERα, Wnt3a, and β-catenin. These results suggested that vibration activated the canonical Wnt/β-catenin pathway in rBMSCs via ERα.

## Discussion

Exercise therapy has been considered the safest and most effective way to strengthen bones to date, and is also an effective measure to prevent PMO, helping to minimize the side effects of treatment [16]. This study showed that LMV prevented PMO in rats, and that the application of LMV in early PMO was more effective than E2.

PMO previously relied on drug therapy. The drugs used for PMO treatment include two categories based on their roles in inhibiting bone resorption and promoting bone formation. Among the approved drugs, only teriparatide (human recombinant parathyroid hormone) promotes bone formation; the others, such as bisphosphonates, denosumab (RANKL monoclonal antibodies), and raloxifene (selective ER modulator) inhibit bone resorption, and cannot fundamentally solve the problem of reduced bone-forming ability in patients. Moreover, although these drugs can delay or stop bone absorption, they usually require long-term use, are expensive, and have many potential side effects and associations, including gastric ulcer, esophageal cancer, atrial fibrillation, atypical femoral fracture, and osteonecrosis of the jaw, making it difficult for patients to accept and adhere to long-term medication [22,23]. In this study, we confirmed that E2 increased the BMD in OVX rats, but induced hyperenlargement of the uterus. LMV not only increased the BMD, but also improved the biomechanical properties of bone in OVX rats without certain side effects, like E2.

High magnitude vibration (regardless of frequency) has been identified as a hazardous factor by the International Organization for Standardization (ISO) [24]. LMV with an acceleration of gravity of less than 1×***g*** was usually used in therapy of osteoporosis. Zhou et al. [12] applied vibration of 0.3×***g*** and 40 Hz for 30 min/12 h and 5 days/week to ovariectomized rats, and found after 12 weeks that vibration can enhance osseointegration by improving microstructure parameters surrounding implants. Li et al. [25] found that vibration of 0.25×***g***, 35 Hz, and 15 min/day for 8 weeks can up-regulate the expression of osteogenesis-related proteins in bone tissues of OVX rats. Qing et al. [26] detected that after 8 weeks of vibration at 0.3×***g***, 30 Hz, and 20 min/day, the deterioration of trabecular bone in OVX rats was ameliorated and the tibia trabecular BMD was significantly increased. However, Brouwers et al. [27] used a vibration of 0.3×***g***, 90 Hz on ovariectomized rats, twice a day for 20 min, 5 days a week for 6 weeks; no changes in structure and strength of tibiae were observed, and the bone structure in rats was still degraded. Xie et al. [28] concluded that vibration of 0.3×***g***, 30 Hz, and 20 min/day for 16 weeks might exacerbate trabecular bone loss in ovariectomized rat femurs. The different effects of vibration on OVX rats might be because of the tested parameters.

In the preliminary experiment, we loaded various parameters on the rats from 12 to 20 weeks including 0.9×***g*** and 90 Hz, 0.9×***g*** and 45 Hz, 0.3×***g*** and 90 Hz, and 0.3×***g*** and 45 Hz, and the results showed that 0.9×***g*** and 45 Hz significantly promoted the bone formation (data now shown). Thus, in the present study, we selected an LMV of 0.9×***g*** and 45 Hz for 20 min everyday, once a day and 5 days a week for 8 weeks (from 12 to 20 weeks postoperatively) or 19 weeks (from 1 to 20 weeks postoperatively). It was demonstrated that the estrogen was significantly elevated in the first 4 weeks after ovariectomy, and then was reduced significantly at 8 or 12 weeks after ovariectomy [29]. Therefore, we did not treat rats with E2 at the first week. We found that LMV increased BMD and bone strength and promoted bone formation of OVX rats significantly better than E2. Moreover, the application of LMV in early osteoporosis was more effective.

BMSCs are stress-sensitive cells that are constantly stimulated by the mechanical environment within the marrow cavity. They play important roles in osteogenic differentiation and bone formation during fracture healing [30]. We demonstrated that LMV promoted the differentiation of rBMSCs into osteoblasts and inhibited their differentiation into adipocytes. This is consistent with the results of Luu et al. [31]. E2 could only promote osteogenic differentiation, but could not inhibit adipogenic differentiation of rBMSCs. These results indicate that LMV is more effective than E2 in promoting the bone formation of rBMSCs.

BMSCs can also secrete OPG, which competitively binds to RANKL, causing RANKL to lose binding activity to RANK, thus inhibiting the generation of osteoclasts [32]. Therefore, the balance between RANKL and OPG determines the generation and activity of osteoclasts. In our study, LMV decreased the ratio of RANKL/OPG in rBMSCs, suggesting that LMV reduced the generation and activation of osteoclasts and reduced bone resorption. This is consistent with the results of Pichler et al. [33] and Lau et al. [34] on bone cells.

Our study found that LMV and E2 caused a significant increase in ERα expression in rBMSCs and promoted the expression of Wnt3a and β-catenin. It was previously reported that ER and Wnt3a can synergistically promote bone formation [35]. These LMV effects could be inhibited by ER inhibitor ICI. Furthermore, ICI inhibited the LMV-promoted differentiation of rBMSCs into osteoblasts. The estrogen pathways in LMV effects should be further studied using shRNA in the future. We did not observe a synergistic effect of E2 and vibration (data not shown).

In summary, LMV promotes osteogenic differentiation, but inhibits adipogenic differentiation of rBMSCs via the canonical Wnt pathway by up-regulating ERα. LMV promotes bone formation with no significant effects on body and uterus weight, which is relatively safer and more effective than E2. In early osteoporosis, LMV can effectively suppress progression.

## Abbreviations

BMD: bone mineral density
Col I: type I collagen
DMEM-LG: Dulbecco’s Modified Eagle Medium, Low Glucose
ER: estrogen receptor
E2: estradiol
ICI: ER inhibitor ICI 182,780
LMV: low magnitude vibration
OCN: osteocalcin
OPG: osteoprotegerin
Osx: osterix
PMO: postmenopausal osteoporosis
PPARγ: peroxisome proliferator-activated receptor γ
qRT-PCR: quantitative real-time polymerase chain reaction
RANKL: receptor activator of nuclear factor-κ B ligand
rBMSC: rat bone marrow-derived mesenchymal stem cell
RIPA: radio-immunoprecipitation assay
RUNX2: runt-related transcription factor 2
Tb.Sp: trabecular separation
TBST: Tris-buffered saline buffer with Tween 20
Wnt: Wingless and Int1
%E-L.Pm: percentage of labeled endosteal perimeter
%P-L.Pm: percentage of the labeled periosteal perimeter
%Tb.Ar: percentage of the trabecular area
